# Molecular characterization of a novel β-defensin isoform from the red-toothed trigger fish, *Odonus niger* (Ruppel, 1836)

**DOI:** 10.1186/s43141-021-00175-6

**Published:** 2021-05-12

**Authors:** S. Neelima, K. Archana, P. P. Athira, M. V. Anju, V. V. Anooja, I. S. Bright Singh, Rosamma Philip

**Affiliations:** 1 Department of Marine Biology, Microbiology & Biochemistry, Cochin University of Science and Technology, Cochin, 682016 India; 2 National Centre for Aquatic Animal Health, Cochin University of Science and Technology, Cochin, 682016 India

**Keywords:** Antimicrobial peptides, β-defensin, Fish, *Odonus niger*

## Abstract

**Background:**

The concern regarding a post-antibiotic era with increasing drug resistance by pathogens imposes the need to discover alternatives for existing antibiotics. Antimicrobial peptides (AMPs) with their versatile therapeutic properties are a group of promising molecules with curative potentials. These evolutionarily conserved molecules play important roles in the innate immune system of several organisms. The β-defensins are a group of cysteine rich cationic antimicrobial peptides that play an important role in the innate immune system by their antimicrobial activity against the invading pathogens. The present study deals with a novel β-defensin isoform from the red-toothed trigger fish, *Odonus niger*. Total RNA was isolated from the gills, cDNA was synthesized and the β-defensin isoform obtained by polymerase chain reaction was cloned and subjected to structural and functional characterization in silico.

**Results:**

A β-defensin isoform could be detected from the gill mRNA of red-toothed trigger fish, *Odonus niger*. The cDNA encoded a 63 amino acid peptide, β-defensin, with a 20 amino acid signal sequence followed by 43 amino acid cationic mature peptide (*On*-Def) having a molecular weight of 5.214 kDa and theoretical p*I* of 8.89. *On*-Def possessed six highly conserved cysteine residues forming disulfide bonds between C1–C5, C2–C4, and C3–C6, typical of β-defensins. An anionic pro-region was observed prior to the β-defensin domain within the mature peptide. Clustal alignment and phylogenetic analyses revealed *On*-Def as a group 2 β-defensin. Furthermore, it shared some structural similarities and functional motifs with β-defensins from other organisms. *On*-Def was predicted to be non-hemolytic with anti-bacterial, anti-viral, anti-fungal, anti-cancer, and immunomodulatory potential.

**Conclusion:**

*On*-Def is the first report of a β-defensin from the red-toothed trigger fish, *Odonus niger.* The antimicrobial profile showed the potential for further studies as a suitable candidate for antimicrobial peptide therapeutics.

## Background

The immune system of vertebrates comprises of adaptive and innate systems and the first line of defence against the invading pathogens is mainly supported by the innate immune system. Antimicrobial peptides (AMPs) are crucial members of this innate immune system, possessing a myriad of defence properties such as anti-microbial, anti-fungal, anti-parasitic, and anti-cancer properties. Their small size, amphipathic and cationic nature, and ability to battle against broad spectrum of infectious agents even at micromolar concentrations has made these natural molecules, potent candidates for antibiotic-alternative therapeutics. These molecules work as synergistic cocktails in vivo and have been explored in combination with conventional antibiotics [1]. The AMP (http://aps.unmc.edu/AP/main.php) database contains over 3180 antimicrobial peptide records. Fishes also possess diverse classes of these host defence peptides such as cathelicidins, hepcidins, piscidins, pleurocidins, histone-derived peptides, and defensins [2].

Vertebrate defensins are classified into three sub-families, i.e., α, β, and θ defensins based on the spatial distribution of conserved cysteine residues that mediate intramolecular disulfide bonding [3]. The discovery of fish defensin was through a database mining approach in fish genomes such as, zebrafish (*Danio rerio*) and the puffer fishes (*Takifugu rubripes* and *Tetraodon nigroviridis*) [4]. Further, it led to the discovery of this AMP class from several marine as well as freshwater fish species like *Oreochromis niloticus,* *Siniperca chuatsi*, *Liza haematochelia*, *Paramisgurnus dabryanus* and *Gerres filamentosus* [5–9].

Defensins reported from fishes belong to β-defensins, marked by a distinctive molecular framework of six conserved cysteine residues, forming three disulfide bonds between C1–C5, C2–C4, and C3–C6 [10]. Moreover, the last two cysteines are consecutively situated in ‘CCXn’ pattern (where *n* ≥ 1) near the C-terminus of the peptide [11]. Potential of fish β-defensins explored so far are antibacterial [12–15], antiviral [16, 17], chemotactic, immunomodulatory, and reproductive regulation [12, 14, 15, 18]. The red-toothed trigger fish are bottom-dwellers and reef-associated organisms. With its striking bluish green hue, it has gained attraction as a marine ornamental fish [19, 20]. This work was aimed at detection of novel isoforms of defensin from marine fishes. A β-defensin gene could be isolated from the red-toothed trigger fish, *Odonus niger* (Ruppell, 1836) and the structural/functional characterization were done in silico to understand the bioactive potential of the molecule for therapeutic applications.

## Methods

### Sample collection

**Fig. 1:**
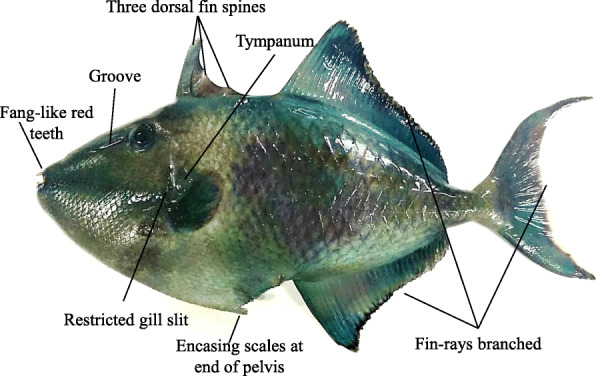
Red-toothed trigger fish, *Odonus niger* used for the study

A red-toothed trigger fish (Fig. 1) was collected from the Cochin estuary (9° 57′ 52.1388′′ N, 76° 16′ 55.8588′′ E) and transported to the lab live under aeration. Identification was done using ‘FAO Species identification sheets for fishery purposes’ (http://www.fao.org/3/ad468e/AD468eBB.pdf). Gills were excised and macerated thoroughly in TRI Reagent® (Sigma-Aldrich) and preserved at – 20 °C in a Freezer (Sanyo, Japan) before RNA isolation.

### Total RNA isolation and cDNA synthesis

Total RNA was isolated using TRI Reagent® (Sigma-Aldrich) following the manufacturer’s protocol and dissolved in 30 μl of DEPC treated water. The purity and quality of RNA was tested using 0.8% agarose gel. Single stranded cDNA was synthesized using RNA that showed *A*_260_:*A*_280_ ≥ 1.8. First strand cDNA was generated in a 20 μl reaction volume containing 5 μg total RNA, 1× RT buffer, 2 mM dNTP, 2 mM oligo d(T20), 20 U of RNase inhibitor, and 100 U of MMLV reverse transcriptase. The reaction was conducted at 42 °C for 1 h followed by an inactivation step at 85 °C for 15 min. Quality of cDNA was tested by PCR amplification of β-actin, using gene-specific primer pair (β-def F 5′gatcatgttcgagaccttcaacac 3′ and β-def R 5′ cgatggtgatgacctgtccgtc 3′).

### PCR amplification and TA cloning

The primers β-def F and β-def R were used for PCR amplification of beta defensin using cDNA as template in a 10 μl reaction volume containing EmeraldAmp® PCR Master Mix and H_2_O. The thermal profile used was 95 °C for 2 min followed by 35 cycles of 94 °C for 15 s, 57 °C for 30 s, and 72 °C for 30 s and a final extension at 72 °C for 10 min. Amplified products were analyzed by 1% agarose gel electrophoresis in TBE buffer, stained with ethidium bromide, and visualized using Syngene G Box Gel documentation unit. The PCR amplicons were then ligated to pGEM®T Easy vector (Promega) and transformed to DH5α *E. coli* competent cells, using manufacturer’s protocol. Transformed *E. coli* were cultured in LB broth (500 μl), at 37 °C with continuous shaking at 150 rpm for 1 h 30 min. This was then plated onto LB agar plates containing Ampicillin (100 μg ml^−1^), X-gal (80 μg ml^−1^) and IPTG (100 mM). Due to insertional inactivation of alpha-peptide subunit of beta-galactosidase enzyme, colonies with positive transformants could be identified by blue/white screening. Thus, white colonies were selected and patched onto fresh LB agar plates and cultured overnight in LB broth containing Ampicillin (100 μg ml^−1^) with continuous shaking at 200 rpm and 37 °C for plasmid isolation.

### Plasmid isolation and sequencing

Plasmid isolation was carried out using overnight grown culture, using GenElute™ Plasmid Miniprep Kit (Sigma) and quality of the plasmid was analyzed on a 0.8% agarose gel. The recombinant plasmids were subjected to PCR amplification using vector-specific and gene-specific primers. The plasmid was sequenced using T7F and SP6R primers on an ABI 3730XL DNA sequencer (Applied Biosystem, USA) at AgriGenome Sequencing Facility, India.

### Sequence analysis and molecular property prediction

Peptide sequence was determined by the ExPASy translate tool (https://web.expasy.org/translate/). The nucleotide as well as the deduced amino acid sequences was subjected to NCBI blast (https://blast.ncbi.nlm.nih.gov/) for similarity search and identification. Signal peptide and pro-peptide were detected using SignalP 5.0 (http://www.cbs.dtu.dk/services/SignalP/) and ProP 1.0 (http://www.cbs.dtu.dk/services/ProP/) [21, 22]. Recognition of the peptide domain was predicted using SMART (http://smart.embl-heidelberg.de/) [23]. The possible disulfide bonds linking the cysteine residues were predicted by CYS_REC tool from Softberry site (http://www.softberry.com/). APD3 (http://aps.unmc.edu/AP/main.php) and ProtPram tool (https://web.expasy.org/protparam/) of ExPASy were used to compute various physico-chemical characteristics of the peptide [24, 25]. Jalview Version: 2.11.1.0 and Mega X applications were used for clustal alignment and phylogenetic analysis respectively [26, 27]. Disorder and surface accessibility were analyzed in RaptorX [28]. 3D structure for the peptide was predicted from the I-TASSER (Iterative Threading ASSEmbly Refinement) suite (https://zhanglab.ccmb.med.umich.edu/I-TASSER/) and the PDB data was visualized in DeepView/Swiss-PdbViewer 4.1.0 (http://www.expasy.org/spdbv/) [29, 30]. TANGO (http://tango.crg.es/) and AGGRESCAN (http://bioinf.uab.es/aggrescan/) were used for in vitro and in vivo aggregation analysis [31–34].

### Bioactive potential prediction

Anti-bacterial, anti-viral, and anti-fungal activities of the peptide were predicted in iAMP pred (http://cabgrid.res.in:8080/amppred/) [35]. Anti-cancer potential of the peptide was forecasted using AntiCP server (http://crdd.osdd.net/raghava/anticp/) [36]. CellPPD (http://crdd.osdd.net/raghava/cellppd/) predicted the cell penetrating probability of the peptide [37, 38]. Immunomodulatory potential was analyzed using PIP-EL web-based server (http://www.thegleelab.org/PIP-EL/) and presence of antigen presenting cell epitope was predicted by Vaxin-PAD server (http://crdd.osdd.net/raghava/vaxinpad/) [39, 40]. The hemolytic activity of the peptide was analyzed in Hemo-PI (http://crdd.osdd.net/raghava/hemopi/) [41]. The I-TASSER suite was used for predicting consensus Gene Ontology (GO) terms for the peptide.

## Results

### Molecular characteristics of the β-defensin from *Odonus niger*

**Fig. 2:**
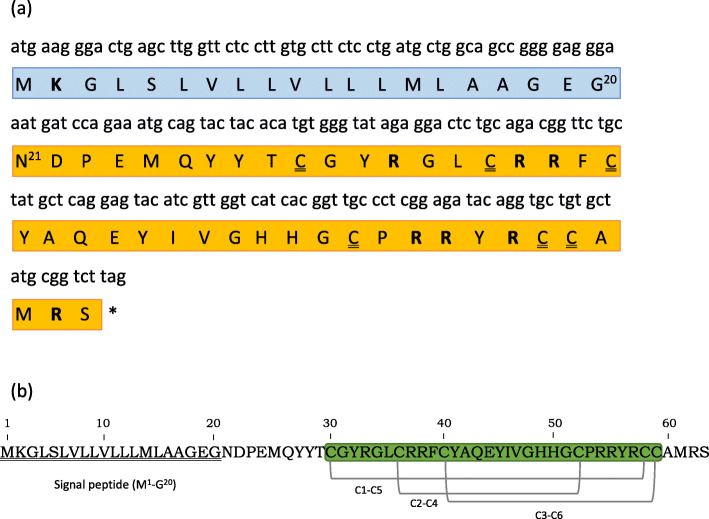
**a** cDNA (192 bp) and deduced amino acid (63 aa) sequences of β-defensin from *Odonus niger*. The blue boxed amino acid sequences (20 aa) denote the signal peptide region with cleavage site predicted in between the Gly^20^-N^21^ residues with SignalP-5.0. The remaining 43 aa residue of mature peptide sequence is in yellow shade. All positively charged residues are shown in bold, and the cysteine residues are double-underlined. ‘*’ denotes termination. **b** An illustration of the SMART and Pfam identified β-defensin domain is shown in green shade (C^30^–C^59^) within which three predicted disulfide bonds of the pattern C1–C5, C2–C4, and C3–C6 are shown

A 192-bp nucleotide sequence encoding 63 amino acids (aa) β-defensin was identified from cDNA of gill sample from the red-toothed trigger fish, *Odonus niger*, hereafter referred to as *On*-Def (Accession No. MT662112) (Fig. 2a). BLASTp displayed 100% query coverage and a maximum identity of 96.83% with β-defensins from *Siniperca chuatsi (*ACO88907.1*)*, followed by 95.24% identity to *Channa striata* (QJW82621.1) and 93.65% each to *Planiliza haematochelius* (AIK66783.1), *Oplegnathus fasciatus* (ADJ21805.1), and *Trachinotus ovatus* (AWY04221.1). The web-resource SMART annotated the β-defensin domain at C^30^–C^59^ of the *On*-Def (Fig. 2b). The Pfam domain analysis in SMART revealed a β-2-defensin domain for the very same region with *e* value of 0.00069 and gave an insignificant Pfam-A match to defensin-beta-2 family protein domain, belonging to the defensin/myotoxin superfamily (clan CL0075). Disulfide bonding pattern C1–C5, C2–C4, and C3–C6, typical of β-defensin was observed for the *On*-Def using the CYS_REC tool. A signal peptide of 20 aa with a cleavage site between glycine and arginine (G^20^-N^21^) residues (Fig. 2a) were observed and no propeptide cleavage site was detected. The molecular weight of the full-length 63 aa peptide was 7.23 kDa with a theoretical p*I* of 8.86. The mature peptide comprised of 43 residues (Fig. 2a), with molecular weight 5.214 kDa and theoretical p*I* of 8.89. The active mature peptide region was arginine (R) rich (16.3%) and the cysteine (C) and tyrosine (Y) residues comprised 14% each. The mature *On*-Def was cationic with a net charge of + 4.5 with positively charged residues (R + K) 7 against the 3 negatively charged residues (D + E). The region between the signal peptide and the first cysteine residue of the β-defensin domain (N^21^-T^29^) had negatively charged residues like aspartate and glutamate. It alone had a predicted anionic charge of − 2.0. Instability index (II) was 39.09 and the ProtParam tool estimated half-life of peptide in mammalian reticulocytes in vitro was 1.4 h; yeast in vivo 3 min and *E. coli* in vivo > 10 h, following the “N-end rule” for predicting half-life of proteins in the three model organisms [42]. The aliphatic index (AI) provides the relative volume occupied by aliphatic side chains as a positive factor for the increase of thermostability of globular proteins, for which the mature *On*-Def recorded 29.53. The grand average of hydropathicity (GRAVY) index displayed − 0.821 and Boman index showed 2.88 kcal/mol.

### Sequence alignment and phylogeny

**Fig. 3:**
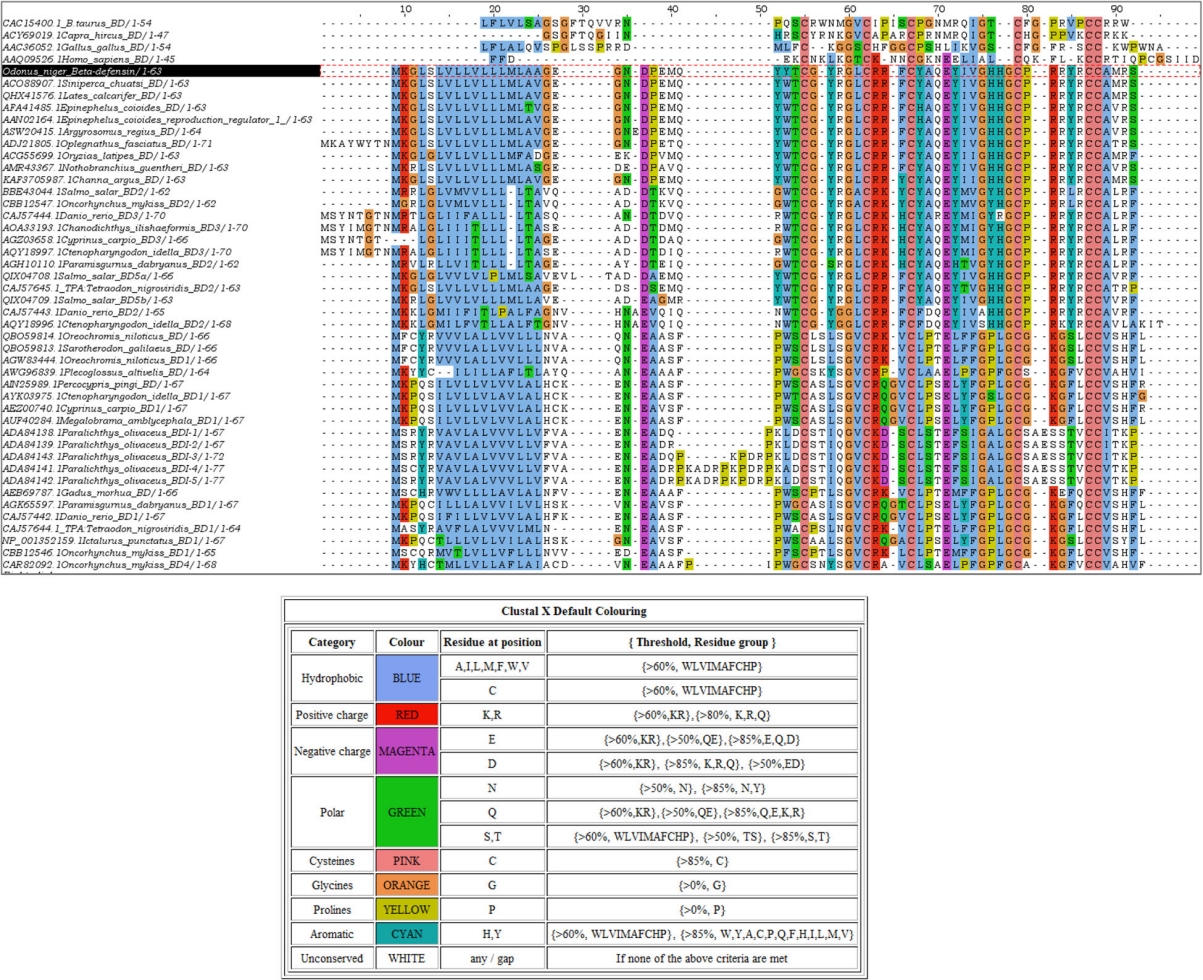
MAFFT alignment of *Odonus niger* β-defensin (*On*-Def) was done against other β-defensins using the Jalview Version 2.11.1.0 workbench. *On*-Def peptide sequence is highlighted. ClustalX colour scheme was applied to the alignment to get a general view of any conservation pattern in the amino acid profile across different sequences

The peptide alignments revealed that the deduced amino acid sequence of β-defensin from the cDNA of *O. niger* was highly similar to other fish β-defensins, and shared six cysteine residues (C^30^, C^36^, C^40^, C^52^, C^58^, and C^59^) at the conserved position of the mature peptide. Moreover, there is a ‘G-X-C2’ motif conserved, which includes the second cysteine residue. The ‘X’ residue of this motif seems to be hydrophobic residue, displayed in blue (Fig. 3), in all the fish β-defensins as well as in *Capra hircus* and *Bos taurus*. *On*-Def also possessed this conserved motif being G^34^L^35^C^36^. Additional to the ‘G-X-C2’ motif, a ‘GXXGC4’ motif common to fish defensins was also present in *On*-Def being G^48^–C^52^. E^44^ in *On*-Def is conserved among all other fish β-defensins as well as in human β-defensin and is the only negatively charged residue within the β-defensin domain. The region prior to C1 residue has conserved negatively charged residue E/D, the same being D^22^ for *On*-Def at the site. The only residue difference in the whole mature peptide region of *On*-Def compared to the most similar *Siniperca chuatsi* β-defensin was Y^28^ residue, which is tryptophan in the *S. chuatsi* β-defensin. Corresponding to this site, hydrophobic residues W/L/A are frequent in other fish defensins. Adjacent to C2, there is an R^37^ residue in *On*-Def. In other fish defensins, positively charged residues R/K are recurrent at the analogous site.

**Fig. 4:**
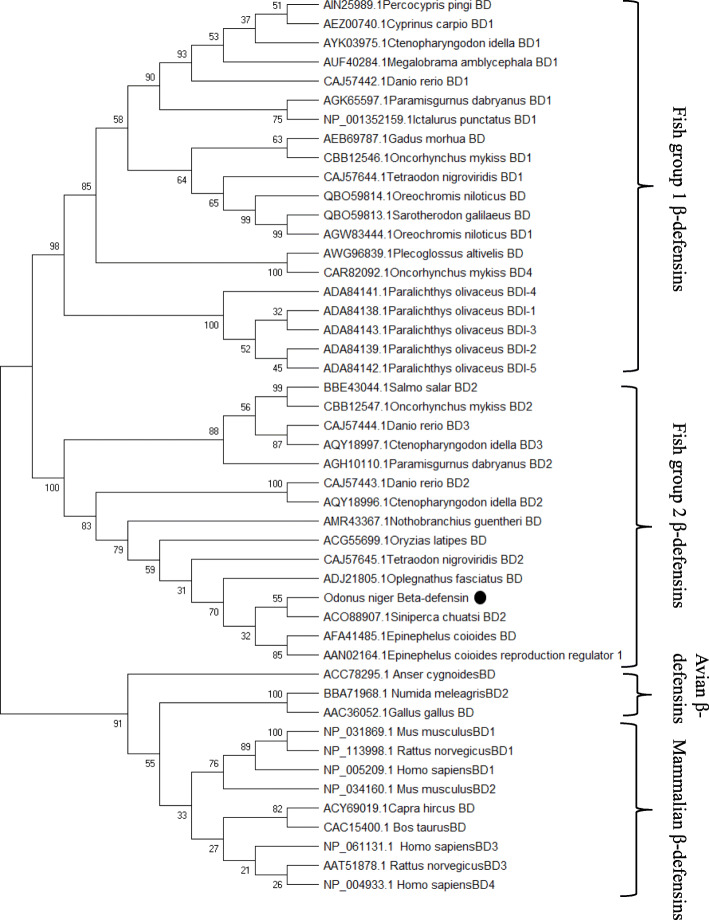
A bootstrapped neighbour-joining tree obtained using MEGA version X, illustrating relationships between the deduced amino acid sequence of *Odonus niger* β-defensin (*On*-Def) with other β-defensins. Values at the node indicate the percentage of times that the particular node occurred in 1000 trees generated by bootstrapping the original deduced amino acid sequences. Mammalian and bird β-defensins are separated as a branch from the fish β-defensin branch. Within the fish β-defensin branch, sub-branching of group 1 and group 2 are visible. *On*-Def is marked as ‘●’, well nested in the fish β-defensin group 2 clade

Two groups of fish β-defensins are evident in the alignment with respect to the number of residues between the first two cysteine residues. *On*-Def is of the group2 β-defensins (that contains fish β-defensin 2/BD2 and β-defensin 3/BD3) that follow the C1-X5-C2 pattern. Moreover, this group has a conserved ‘PRRY/LR’ motif between C4 and C5, which is also seen between C^52^ and C^58^ of *On*-Def. The other group, i.e., group 1 fish defensins, have six residues that stretch between the first two cysteines (C1-X6-C2) and they do not have PRRY/LR motif. The overall similarity of group 2 fish β-defensins is also evident from the phylogenetic tree (Fig. 4), where all bony fish β-defensins clustered as a branch, away from the mammalian and bird β-defensins. Within the fish β-defensin group, two sub-branches are formed (group 1 and group 2). *On*-Def is well nested within the fish defensin group 2 clade, showing similarity to the *S. chuatsi* β-defensin.

**Fig. 5:**
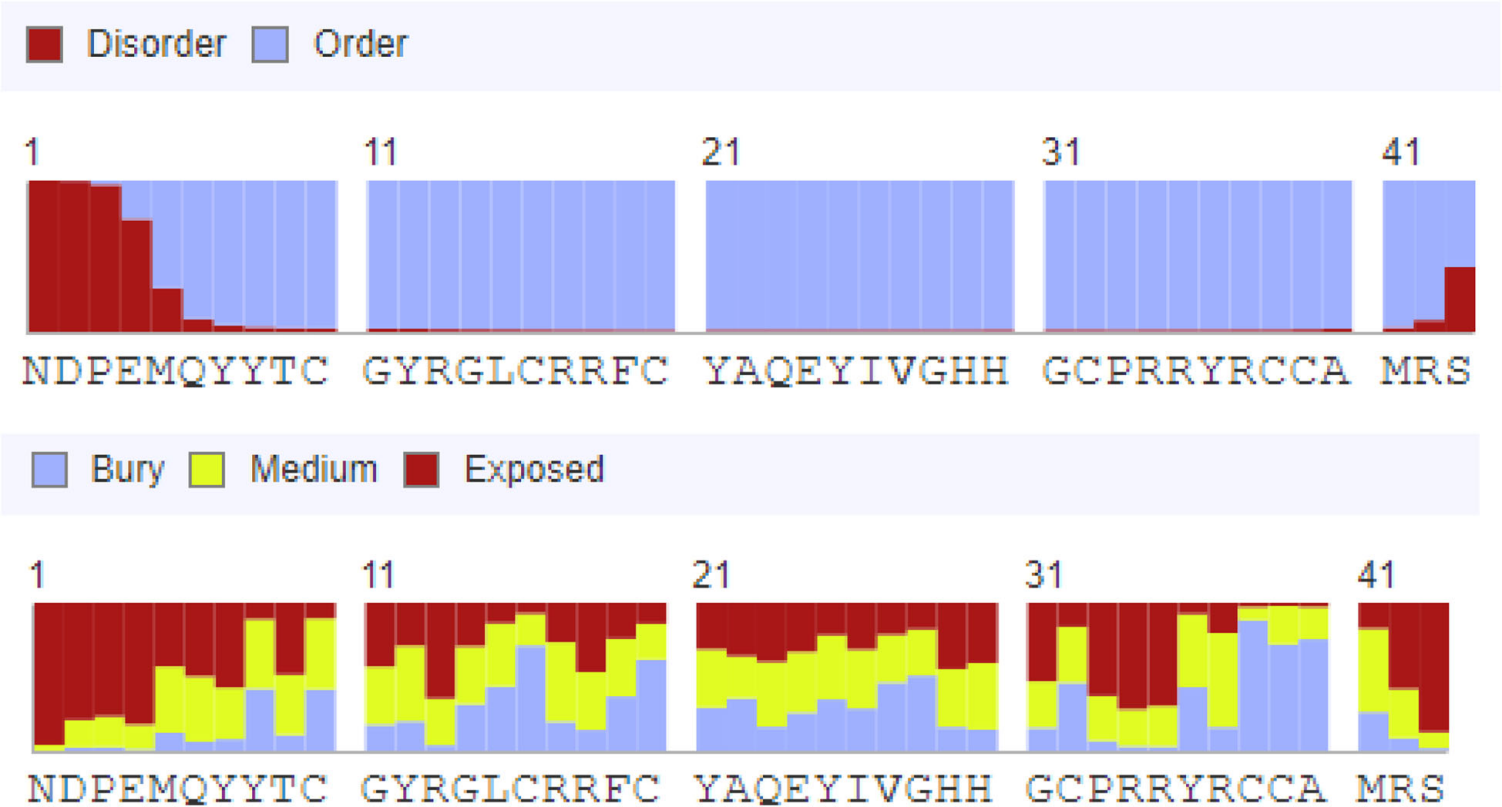
RaptorX forecasted N-terminal region of the *On*-Def mature peptide as disordered. Most of the molecule was seen exposed showing surface accessibility

RaptorX predicted the peptide to have lesser buried residues and more of exposed pattern (41% exposed, 37% medium exposure, 20% buried), denoting greater interaction with solvent. Four residue (9%) positions of N-terminus were predicted as disordered (Fig. 5).

### Structure of On-Def

**Fig. 6:**
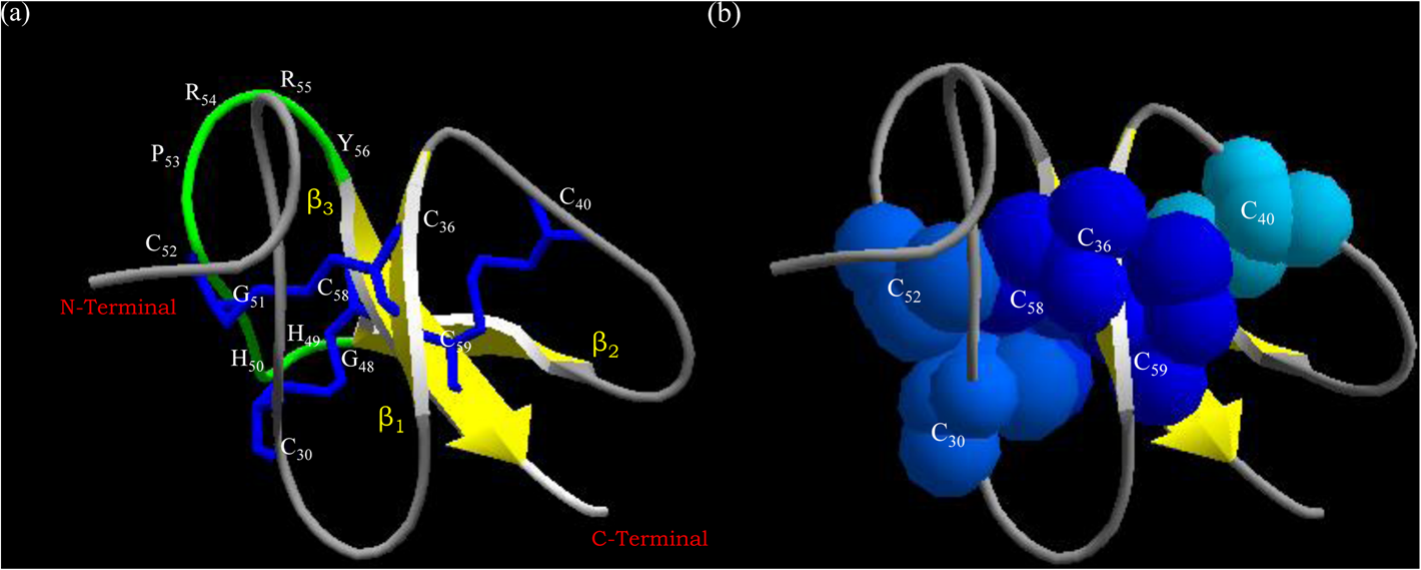
**a** Predicted β-defensin domain model for *Odonus niger* β-defensin (*On*-Def) by I-TASSER. The three β-strands are denoted yellow with the three disulfide bonds (blue) stabilizing the β-defensin fold. The C-terminal β-strand (β3) is trapped between the other two β strands (β1 and β2) by the β-hairpin G^48^–Y^56^ (green) forming the typical anti-parallel β-defensin structure. **b** The buried position of cysteine residues with their disulfide bonds were computed using the accessibility color tool of Swiss PDB Viewer 4.1, which displays accessibility of residues ranging from deep blue (most buried) to red (most exposed)

Out of the five models predicted by I-TASSER, the model with highest C-score of − 2.35 was chosen (C-score range lies between − 5 and 2) and viewed in the SwissPDB Viewer 4.1. The β-defensin domain of *On*-Def mature peptide possessed three anti-parallel β-sheets stabilized by the three disulfide bonds formed by Cys^30^–Cys^58^ (C1–C5), Cys^36^–Cys^52^ (C2–C4), and Cys^40^–Cys^59^ (C3–C6), typical of the β-defensin domain fold (Fig. 6a). The accessibility of residues computed in SwissPDB Viewer displayed all the cysteines forming the disulfide bonds to be in a buried position (Fig. 6b).

### Aggregation propensity prediction

**Fig. 7:**
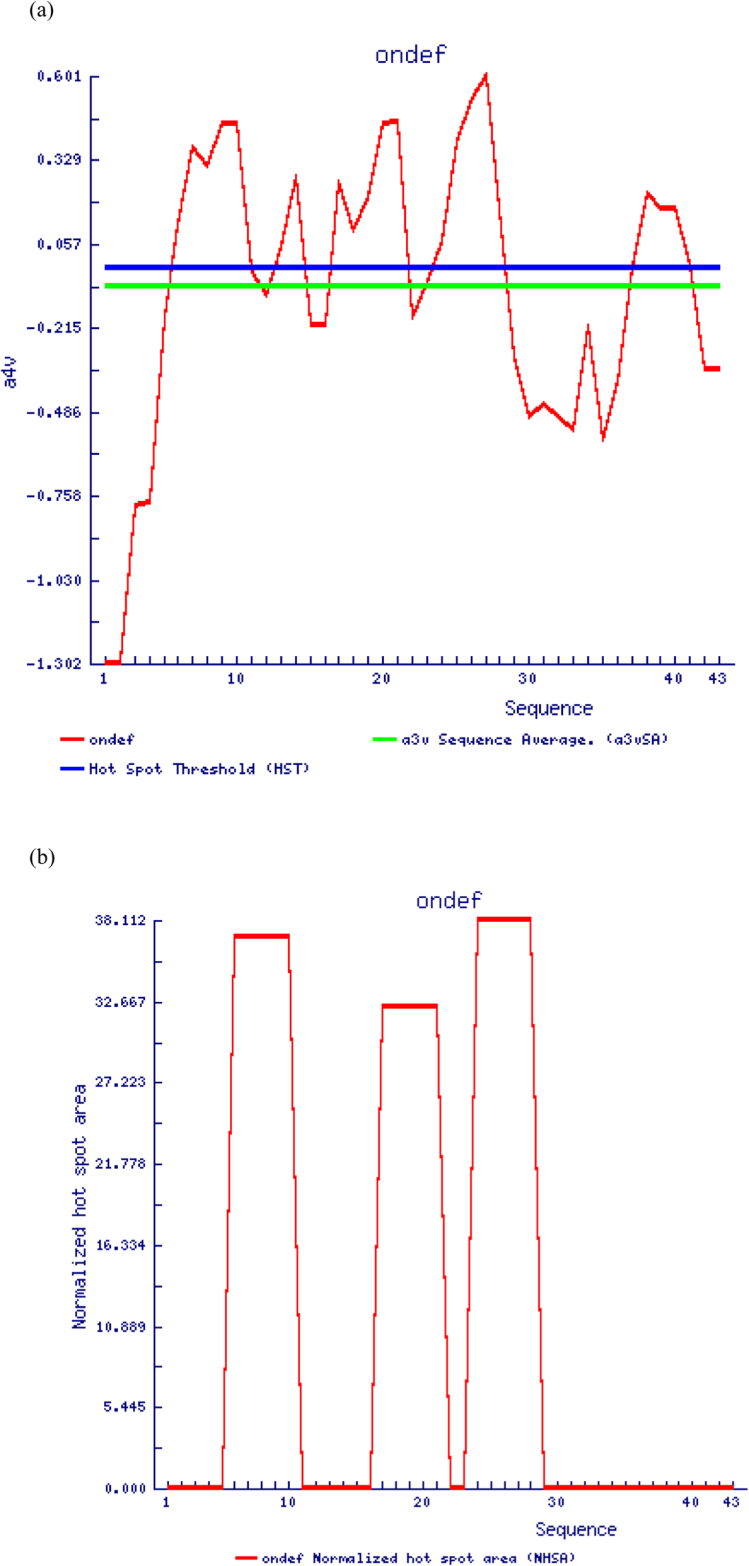
**a** Graph illustrating the aggregation tendency profile of every introduced sequence in the *On*-Def mature peptide. The amino acid aggregation propensity value a3v (green line) is below the hot-spot threshold HST (blue line) in the graph, denoting the presence of fewer aggregation prone residues. **b** Graph depicting exclusively the area occupied by the residues involved in a hot spot (normalized by the peptide’s length). Regions 6–10, 17–21, and 23–29 of the mature *On*-Def sequence are predicted as potential aggregation regions

TANGO and AGGRESCAN algorithms were used to predict in vitro (or “in solution”) and in vivo aggregations respectively for the mature peptide *On*-Def. TANGO analyzed both the beta-sheet and alpha-helix aggregation in terms of Agg and Helagg parameters, respectively. For *On*-Def, Agg, and Helagg, values were 0, implying no in vitro aggregation tendency for the peptide. In case of AGGRESCAN, the global measure of the protein aggregation propensity (Na4vSS) was − 8.50, denoting a very low or least aggregation tendency. This is evident from the graph showing aggregation tendency profile of every introduced sequence (Fig. 7a). However, aggregation hotspots were identified by the software (Fig. 7b).

### In silico activity prediction and Gene Ontology annotation

Bioactive potential of the mature *On*-Def was predicted using a series of web-based servers. Most of these online platforms are based on support vector machine (SVM) algorithm for prediction, where the probability score is between 0 and 1. Values > 0.5 were considered as positive and < 0.5 as negative, and the higher the score, the greater the probability for having bioactive potential. *On*-Def was predicted to have anti-bacterial, anti-viral, anti-fungal, and anti-cancer activity (Table 1). It did not show any cell penetrating potential when tested against the datasets of cell penetrating peptides in the CellPPD server. Peptides have potential to act as vaccine adjuvants as they have immunomodulatory potential to activate innate immune system. The PIP-EL server predicted pro-inflammatory potential for the peptide. Moreover, the *On*-Def mature peptide was screened for potential antigen presenting cell epitope regions in the VaxinPAD server with default fragment length chosen for scanning as 10 residues and SVM threshold set to 0.5. About 34 fragments were predicted out of which the highest SVM score obtained was 0.98 that corresponded to the residue region ‘GHHGCPRRYR’ of the mature *On*-Def. This 1.23 kDa fragment had a net charge of + 3 with a low hydropathy value of − 0.66. *On*-Def was non-hemolytic as per the HemoPI.

**Table 1:** Probability scores predicted based on SVM algorithm of the respective web-based servers

Server	Probability scores
**iAMPpred**	
Anti-bacterial activity	0.97
Anti-viral activity	0.86
Anti-fungal activity	0.96
**AntiCP**	
Anti-cancer activity	0.75
**PIP-EL**	
Pro-inflammatory potential	0.69
**HemoPI**	
Hemolytic activity	0.20

Consensus GO-terms were annotated for the peptide in the I-TASSER suite, that describe gene products in terms of their associated biological processes, cellular components and molecular functions (Table 2). The predicted annotations of the three categories of Gene Ontology showed GO-scores ranging between values 0.12 and 0.38. Among them, the terms annotated for biological processes revealed that the peptide is related to processes involving antimicrobial activity and regulation. The cellular component annotation denoted that the peptide is released extracellularly for its biological action.

**Table 2:** Consensus GO-term annotations of *On*-Def by the I-TASSER suite. The GO-score associated with each prediction is defined as the average weight of the GO term

GO category	GO-term	Description	GO-Score
Biological process	GO:0002816	*Regulation of biosynthetic process of antibacterial peptides active against Gram-positive bacteria*	0.34
	GO:0006963	*Positive regulation of antibacterial peptide biosynthetic process*	0.34
Cellular component	GO:0005576	*Extracellular region*	0.38
Molecular function	GO:0003723	*RNA binding*	0.12
	GO:0004146	*Dihydrofolate reductase activity*	0.12
	GO:0050661	*NADP binding*	0.12

## Discussion

Aquatic organisms thrive in a microbe rich environment and depend mainly on the first line defence, i.e., the non-specific innate immune system to fend off pathogens. The antimicrobial peptides constitute an important component of the innate immunity and play a crucial role in defence. Works on defensins are comparatively limited and so far only β-defensins have been identified in fishes [2, 10]. In teleosts, the gut, skin, and the gills are the main mucosal surfaces and immune barriers [43]. In the present study, we have identified a β-defensin isoform obtained from gills of the red-toothed trigger fish, *Odonus niger*, and performed its molecular and functional characterization in silico.

Fish defensins generally vary between 60 and 77 amino acids [2], with signal peptide of 18–24 amino acids and a mature peptide spanning 38–45 residues [4]. In Nile tilapia β-defensin, the SignalP predicted mature peptide was shorter by two amino acids at the N-terminal region compared to the actual mature peptide analyzed by mass spectrometry (MS). The computed and observed mature peptides showed comparable bioactivity. However, the actual peptide showed greater activity against two bacterial pathogens [5]. The signal peptide of *Odonus niger* β-defensin (*On*-Def) comprised 20 amino acids (cleavage sites G^20^–N^21^), and the mature peptide region 43 amino acids with a molecular weight of 5.214 kDa and p*I* 8, indicating its cationic nature. β-defensin, *On*-Def, showed 96.83% similarity with β-defensin from *Siniperca chuatsi* (ACO88907.1) [6].

**Fig. 8:**
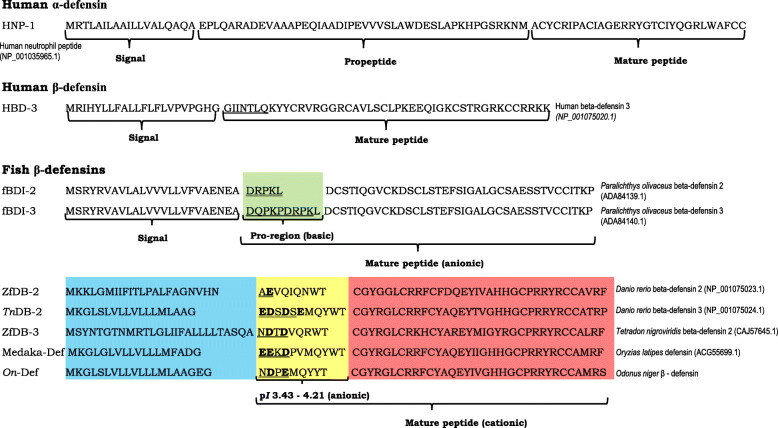
Comparison of pro-regions (yellow) between signal peptide (blue) and β-defensin domains (red) of various cationic fish-defensins. Anionic residues are marked bold and disordered regions underlined. The basic pro-pieces (green) of two anionic olive flounder β-defensins with disordered regions (underlined) are shown. The disordered regions of HBD3 is underlined

β-defensins are processed to their mature forms by signal peptidase cleavage. However, α-defensins require a further enzymatic cleavage to process propeptide to its mature peptide form [44]. The role of a well-defined pro-piece in the human α-defensin HNP1 (Fig. 8) was found to be neutralization of cationic property and thereby preventing auto-cytotoxicity during its synthesis and processing [45]. Similar observation has been made for anionic β-defensins (fBDI-2, fBDI-3) of olive flounder (*Paralichthys olivaceus*) which had pro-like-regions of varying lengths within the mature peptide (Fig. 8). In the olive flounder defensins, these ‘pro-regions’ are very basic and cause neutralization of the anionic mature fBDI [13]. The region between the signal peptide and the predicted β-defensin domain (N^21^–T^29^) in the mature *On*-Def contained anionic residues, D^22^ and E^24^, responsible for a − 2.0 charge and p*I* of 3.67 denoting its anionic nature. Furthermore, the N-terminal residues (N^21^–E^24^) of mature *On*-Def are predicted as disordered regions of the peptide. Disordered regions, due to their remarkable conformational flexibility have been known to play important functional roles in peptides [46]. In human beta-defensin 3 (HBD3), the disordered N-terminal region was found in the helix-capping motif responsible for α-helix integrity to provide high NaCl resistance to the peptide [47]. In mature *On*-Def also, the anionic ‘pro-like-region’ with the predicted disordered nature was found (Fig. 8). When compared with other fish defensins, the cationic fish defensins were found to possess anionic residues as well as disordered regions as predicted by RaptorX. The basic pro-pieces (p*I* 8.75 and 8.59) of two anionic olive flounder β-defensins were also predicted to contain disordered regions. Whether these sequences in the fish defensins influence antimicrobial activity or protect the cells from cytotoxicity requires further investigation.

Instability index (II) was 39.09, classifying the protein as stable molecule in vitro (II > 40 denotes unstable molecule in vitro) [48]. Aliphatic index (AI) of 29.53 symbolized a low value when compared to AIs of proteins from thermophilic bacteria averaging 65–105 [49].

This infers that the peptide may not be much stable for a wide temperature range. GRAVY index of − 0.821 implied that the peptide is likely to associate better with water [50]. Greater proportion of exposed residues and lesser buried residues in *On*-Def also support this polar nature. High Boman index indicated high binding property with other proteins or peptides [51].

Clustal alignment revealed the conserved cysteine residues (six) typical of the β-defensin domain to be present in *On*-Def also, with the last two cysteines consecutively situated, following CCXn pattern of β-defensins, where *n* ≥ 1 near the C-terminus [11]. Other residues that followed a > 80% conservation (observed by percentage identity colour scheme in Jalview) were G^34^ located between the first two cysteines, R^37^ between second and third cysteines, and E^44^ and G^48^ between the third and fourth cysteines. This pattern was first observed in β-defensins identified via computational search strategy to identify 23 human and 48 mouse β-defensins [52]. But this form is not strictly observed for several other β-defensins from humans (HBDs) or the avian group. In them, a glycine is often present, two residues upstream of C2 and two residues upstream of C4. Fish defensins possess a glycine just one residue upstream to C4 [53]. *On*-Def also had a similar ‘G^51’^ adjacent to the fourth cysteine. The functional importance of these conserved residues is yet to be understood.

Among the fish defensins, two groups of β-defensins are obvious, where one group had five residues and the other had six residues between the first two cysteines. *On*-Def belonged to the group with five residues between its first two cysteines, C^30^ and C^36^, denoting that it belongs to group 2 β-defensin [4, 6]. Additionally, the highly basic ‘PRRY(L)R’ motif of group 2 β-defensins was confirmed in *On*-Def between fourth and fifth conserved cysteines. Interestingly, a similar motif ‘PRRYK’ is found adjacent to the third cysteine residue of human beta-defensin 2 (HBD2). The ‘RRYK’ residues of this motif are involved in chemotaxis via HBD2 and glycosaminoglycans (GAG) complex formation on cell surfaces leading to the increase in local concentration of HBD2 molecules that could be presented to CCR6 receptors [54]. The basic residues like lysine and arginine of this motif were found to bind with phosphatidylinositol 4,5-bisphosphate (PIP_2_) mediating the membrane permeabilization in the fungal pathogen *Candida albicans* [55]. The role of this motif is yet to be unravelled in fishes. *On*-Def also showed greater clustering of cationic residues especially towards the C-terminal region as usually observed in β-defensins.

Differences are observed among β-defensins in the N-terminal regions indicating specificity with respect to their functions [11]. Some display alpha-helix in N-terminal region [14], but others possess a coiled region [4]. The β-defensin domain of *On*-Def was held compact by the three disulfide bonds: C^30^–C^58^ connecting coil region before the β1 strand with β3 strand, C^36^-C^52^ connecting β1 and hairpin region between β2 and β3 and finally, C^40^–C^59^ connecting β3 and loop region between β1 and β2 following the C1–C5, C2–C4, and C3–C6 disulfide bridge pattern of β-defensins [56, 57]. The characteristic feature of the disulfide bonding does not seem to be an indispensable factor for antimicrobial activity of β-defensins. Antimicrobial activity of HBD-3 is independent of the presence or absence of disulphide bridges; however, its chemotactic activity was lost in the absence of the disulfide bridges [58]. Studies showed that the ability of β-defensins to interact with microbial membranes is not much dependent on the three-dimensional structure or the disulfide pattern of β-defensins. Instead, it is more based on the ratio of polar to hydrophobic residues distributed within the peptide or its surface and the net positive charge that they carry [58–62]. The cysteines with disulfide bonds are in a buried state towards the centre of *On*-Def, as generally observed in β-defensin family of peptides [11].

The G-X-C4 motif has been found to be conserved in classical defensins as well as β-defensins and is proposed to help dimer assembly in bovine neutrophil β-defensin-12 (BNBD-12) [63] as well as human neutrophil peptide defensin-3 (HNP-3) [64]. The role of this motif has been attributed to formation of an atypical classic type β-bulge, which is probably responsible for forming a twist in the β-sheets, essential for the proper folding [65]. Whether the ‘G-X-C2’ motif in *On*-Def and other fish defensins contribute to such roles is yet to be analyzed. ‘GHHGC’ and ‘PRRY’ conserved motifs formed the β-hairpin region between β2 and β3 strands of *On*-Def. It has been observed in bovine β-defensin-2 (BNBD-2) that hairpin conformation regions with prolines and glycines, play important role in bacterial membrane interaction and this region is rich in charged residues which support electrostatic interactions essential for membrane destabilization [66]. *On*-Def, like other group 2 β-defensins, is rich in arginine residues in this β-hairpin region, proposing a similar role. Arginine is capable of strong electrostatic interaction with multiple lipid head groups due to the charge delocalization over the guanidium group, and capable of causing even trans-membrane pores of lipid bilayers [67, 68]. The P^53^ residue may be important in β-hairpin formation as observed for bovine β-defensin-2 (BNBD-2) [66]. *On*-Def showed 93.65% identity to Lhβ-defensin of Soiny mullet. In Lhβ-defensin, a dimeric structure was predicted, which was mainly stabilized by the van der Waals contacts and cationic-π interactions that enabled positively charged surface to be more exposed. This enhanced its interaction with negatively charged bacterial membranes [7]. Structural similarities to Lhβ-defensin imply similar antimicrobial mechanisms for *On*-Def also.

The aggregation of AMPs is promoted in hydrophobic environment like the bacterial cell membrane and minimum in solution prior to membrane level aggregation [69]. The Shai–Matsuzaki–Huang model explains the activity of antimicrobial peptides which proposes that the peptide interact with anionic lipid head groups of microbial membrane components, displacing the membrane lipids, and thus altering its structure. In some cases, the peptide enters the cytoplasm of the target cell [70]. The POPG membrane disruption leading to water translocation by Lhβ-defensin provides support for this mechanism on behalf of the fish group 2 β-defensins [7]. In the case of *On*-Def, in vivo aggregation was very less and in vitro aggregation was absent with respect to the prediction by AGGRESCAN and TANGO, respectively. However, aggregation prone segments were predicted for *On*-Def mature peptide, probably the regions that are involved in aggregation on bacterial membranes.

Fish β-defensins have been reported to show anti-Gram negative bacterial activity [12, 13, 18, 53], anti-Gram-positive bacterial activity [8, 14, 15, 71], anti-viral activity [16–18, 72], immunomodulatory and chemotactic responses [12, 14, 15, 73], and anti-inflammatory roles [74]. Besides the afore-mentioned bioactivities, *On*-Def is also predicted to have anti-fungal and anti-cancer property. An interesting prediction was the ‘GHHGCPRRYR’ fragment as an antigen presenting cell epitope region by the VaxinPAD server. The consensus GO term annotations predicted the peptide to be possibly involved in pathways leading to defence responses against microbial pathogens.

## Conclusion

Computational predictions revealed the potential of this arginine rich, cationic *Odonus niger* group 2 β-defensin, *On*-Def as a potential antimicrobial agent. *On*-Def with its overall cationic charge and lesser hydrophobicity with aggregation hot-spot regions could be involved in bacterial membrane disruption. Also, there is possibility for oligomeric assembly leading to higher activity/interaction with other immune-related peptides based on higher Boman index predicted. The peptide showed anti-microbial, anti-cancer, and immunomodulatory potential in silico, revealing its credentials as a potential candidate molecule for further studies in vitro leading to its therapeutic applications.

## Data Availability

The data generated during and/or analyzed during the current study are not publicly available (exception is GenBank accessions) but are available from the corresponding author on reasonable request.

## Abbreviations

AMP: Antimicrobial peptide
*On*-Def: *Odonus niger* β-defensin
DEPC: Diethyl pyrocarbonate
II: Instability index
AI: Aliphatic index
GO: Gene ontology
